# Developing a ‘neighbourhood health service’: ensuring equity of access for rural populations

**DOI:** 10.3399/BJGP.2026.0054

**Published:** 2026-03-01

**Authors:** Samuel P Trethewey, Christopher E Clark, Rory Honney, Tamsin Newlove-Delgado

**Affiliations:** 1 Gloucestershire Hospitals NHS Foundation Trust, Gloucester, UK; 2 Children and Young People's Mental Health Research Collaboration (ChYMe), Department of Public Health and Sports Sciences, University of Exeter Medical School, Exeter, UK; 3 Exeter Collaboration for Academic Primary Care (APEx), Department of Health and Community Sciences, University of Exeter Medical School, Exeter, UK; 4 Corner Place Surgery, Paignton, UK; 5 Torbay and South Devon NHS Foundation Trust, Torbay Hospital, Torquay, UK

Recent discourse highlights important conceptual and practical challenges in defining and developing a ‘neighbourhood health service’.^1^ A key part of the Government’s 10-year health plan for England centres around integration and localisation of NHS services to neighbourhoods via so-called neighbourhood health centres, to support its vision for a shift from ‘hospital to community’.^2^

Integrating and localising services to neighbourhoods is a laudable goal, to increase accessibility, strengthen community involvement in design and delivery, and enable greater tailoring and responsiveness to community needs via care that is ‘delivered closer to home’.^3^ But what exactly is a neighbourhood in this context? Is this just renaming and/or regrouping of existing geographically bounded health systems, like primary care networks, without meaningful changes to underlying system structure, strategic oversight, and service delivery?

*“How, then, do we ensure equitable access across different settings, with a level of quality control that reduces postcode lotteries?”*

## What is a neighbourhood?

Conceptually, what is considered a ‘neighbourhood’ varies. There are the physical characteristics (houses, roads, services) and the social context – the people. Contemporary definitions are predictably complex and largely drawn from urban-centric research.^4^ In urban environments, neighbourhoods could simply be a collection of homes on one or more streets but may expand to encompass larger areas and communities.

Defining neighbourhoods in sparsely populated rural contexts, however, is even more ambiguous. The word ‘neighbour’ appears to originate from the Old English word *neahgebūr* meaning ‘near dweller’, which captures the core of the neighbourhood concept well in terms of social and spatial proximity.^5^ Clearly, this is problematic in sparsely populated rural areas, where you might only have a handful of neighbours within walking distance, or none at all. The rural context therefore complicates traditional notions of ‘neighbourhoods’.

## Centralisation versus localisation of services

The 10-year plan proposes a population catchment of ~50 000 people per neighbourhood.^2^ The inevitable geographical and social heterogeneity at this scale brings significant challenges to those looking to design and implement neighbourhood health centres, particularly across large rural areas. For example, in Devon we face considerable challenges around implementation of integrated hub-based models of service delivery, due to our sparse population with inequitable access to public transport, especially for rurally isolated and deprived communities.

This creates tensions between integrating services into centralised hub-based models versus more localised community-based models with smaller geographical footprints which, by definition, must be higher in number to ensure population coverage. A higher number of multidisciplinary health centres poses potential issues with fragmentation and economies of scale. Conversely, more centralised models carry different risks including increased travel distances for rural patients, reducing access rather than increasing it, alongside decreased continuity of care.^6^

For areas with dispersed populations, it may not be feasible to achieve 50 000-person ‘neighbourhoods’ within manageable geographic footprints. Crucially, these issues will be even more prominent for more deprived and disadvantaged rural communities. How, then, do we ensure equitable access across different settings, with a level of quality control that reduces postcode lotteries?

## How can we address these challenges?

These are not new issues facing rural communities and healthcare commissioners, but the Government’s renewed focus on localisation brings it to the fore. Yet, there is not a single mention of the word ‘rural’ in NHS England’s Neighbourhood Health Guidelines.^7^ Also, while the 10-year plan does nod to some of the challenges facing rural health systems, it does not offer any tangible recommendations to address them.^2^

Much, we suspect, will be said of the potential for digital technologies to bridge the gap in access to services for those living in rural and remote communities. Indeed, the shift from ‘analogue to digital’ is another key strategic focus for the government in the 10-year plan.^2^ While digital technologies have an important role in increasing access to support in rural contexts,^8^ they are not a panacea. Many patients will likely still want and need to interact with healthcare professionals via face-to-face services.^9^

We must address ongoing issues with digital exclusion, including via targeted ‘digital facilitation’ programmes and interventions that address structural barriers,^10,11^ or we risk exacerbating inequities. The coincidence of older age populations, lower availability of high-speed broadband, and poorer mobile network coverage in rural settings makes digital exclusion a particular challenge for rural health systems.^12^

Greater collaboration is needed between system leaders and commissioners of healthcare services in rural areas across the country. While it is crucial to recognise rural contexts are heterogenous, so will have different needs, similarities do exist that could benefit from sharing of learning and expertise to overcome mutual challenges.

Looking beyond traditional NHS service provision and strengthening existing community health assets is key, particularly in rural and remote contexts. The Neighbourhood Health Guidelines do recognise the importance of collaboration with wider system partners, but the guidance deprioritises this against other aims. Focusing efforts on ‘connecting communities and making optimal use of wider public services’ will simultaneously support progress towards achieving the central goal to ‘prevent people spending unnecessary time in hospital or care homes’.^7^

It is positive to see mention of the role of voluntary sector services in the 10-year plan as part of its vision to create a holistic neighbourhood health service offer,^2^ but this needs to be meaningfully translated into action. More explicit practical support and guidance for voluntary sector partners is needed to facilitate effective integration into neighbourhood health centres, particularly in rural contexts.

## Concluding remarks

Careful consideration is needed to ensure equity of access to services across different contexts, to avoid increasing inequities for rural and remote populations. At the very least, we need to see explicit recognition of these issues by those involved in coordinating and leading the roll-out of neighbourhood health centres. In addition, greater investment in, support for, and involvement of practitioners and policymakers with rural health system expertise and experience is crucial to realise the vision of a ‘neighbourhood health service’.
